# Combination of Curcuminoids and Acupressure for Inflammation and Pain in Older People with Osteoarthritis Genu: Protocol for a Randomized Controlled Trial

**DOI:** 10.2196/54970

**Published:** 2024-06-24

**Authors:** Srinalesti Mahanani, Nyoman Kertia, Ema Madyaningrum

**Affiliations:** 1 Doctorate Program of Medical and Health Science Faculty of Medicine, Public Health, and Nursing Universitas Gadjah Mada Yogyakarta Indonesia; 2 Department of Nursing STIKES RS Baptis Kediri Kediri Indonesia; 3 Department of Internal Medicine Faculty of Medicine, Public Health, and Nursing Universitas Gadjah Mada Yogyakarta Indonesia; 4 Dr. Sardjito General Hospital Yogyakarta Indonesia; 5 Department of Mental and Community Health Nursing Faculty of Medicine, Public Health, and Nursing Universitas Gadjah Mada Yogyakarta Indonesia

**Keywords:** osteoarthritis, acupressure, curcuminoids, endorphins, biomarkers, genu, older people, randomized controlled trial

## Abstract

**Background:**

Curcuminoids and acupressure have beneficial effects in reducing pain and inflammation in patients with osteoarthritis. However, only a few clinical trials are investigating biomarkers to prove this objectively.

**Objective:**

This study aims to investigate the effect of acupressure and curcuminoids on inflammatory markers and pain in older people with osteoarthritis genu.

**Methods:**

A randomized controlled trial (RCT) was conducted among older people with osteoarthritis. All participants were randomized to a group that received 30 mg of curcuminoids in turmeric extract capsules and acupressure (group 1) or a group that received a placebo and sham acupressure (group 2) for 3 weeks.

**Results:**

The study was approved by the research ethics board; ClinicalTrials.gov reviewed this protocol. The extracts were manufactured from May 2023 to June 2023. Participant recruitment was conducted in September and October 2023; a total of 72 participants aged 60 years or older participated, of whom 75% (n=54) were female. Data were analyzed in April 2024, and dissemination of results is expected by the end of 2024.

**Conclusions:**

Primary outcomes were assessed at baseline and after the intervention. Relationships were assessed with inflammatory markers, endorphin hormones, and blood level of cycloxygenase-2 hormone. Additionally, secondary outcomes included pain, ability to perform activities of daily living, and quality of life. The beneficial effects that may be found in this trial may be exceptionally relevant in clinical practice, justifying this scientific inquiry. The benefits of herbs and acupressure can be helpful as additional options in treating inflammation and pain in patients with osteoarthritis.

**Trial Registration:**

ClinicalTrials.gov NCT06105840; https://clinicaltrials.gov/study/NCT06105840

**International Registered Report Identifier (IRRID):**

DERR1-10.2196/54970

## Introduction

### Background

One of the degenerative processes that occurs in older people involves the musculoskeletal system. Deterioration includes bone loss and decreased joint fluid volume, which are exacerbated by the body bearing its own weight and can cause older people to experience pain [1]. The experience of prolonged pain in patients with osteoarthritis is considered unendurable and reflected in the common cliché, “reaching the breaking point,” which describes the experience and process of living with unremitting pain; limitations in mobility, leisure, and social activities; and the resulting negative consequences for a patient’s physical and psychological well-being [2]. Patients with osteoarthritis often seek some turning point for positive changes in their quality of life and relief from the incessant pain. The development of complementary nursing knowledge continues to be carried out, including in the energy and biofield therapies and biologically based therapies groups, which are types of complementary alternative medicine (CAM). People aged 50 years and older are likely to be using CAM. Common use of CAM as a complement to conventional medicine—and the high use of multiple prescription drugs—further underscores the need for health care providers and clients, patients, and families to have an open dialogue to ensure safe and appropriate integrated health care [3]. One common therapy practiced in many Asian communities, especially the Javanese, is acupressure, a traditional development of massage. Historically, massage is an action that people often do independently that can act psychologically to make patients feel relief and comfort, but research needs to be conducted empirically to ensure its effectiveness.

The pain experienced by patients with osteoarthritis affects many areas of their quality of life, including physical function, emotional behavior, and mental health. Osteoarthritis-related pain is a significant factor in poor quality of life. The most common pharmacological treatment to control pain is nonsteroidal anti-inflammatory drugs, but these drugs have a considerable risk of causing side effects [4]. Limitations associated with pharmacological treatment result in patients choosing commonly available alternative therapies for pain management. Popular alternative therapies include herbal therapy, therapeutic touch, relaxation techniques, music therapy, acupuncture, and acupressure. Unlike the use of drugs, these alternative therapies do not have dangerous side effects [5].

Research concerning the effectiveness of herbal therapy in patients with inflammatory osteoarthritis was conducted in Indonesia by Kertia [6] among 80 patients. The results showed that administration of curcuminoids extracted from turmeric rhizomes significantly suppressed the activity of synovial fluid monocytes to secrete cyclooxygenase-2 (COX-2) and reactive oxygen intermediates; it also reduced leukocyte numbers and fluid malondialdehyde levels. Synovia reduces osteoarthritis-related joint pain, with an ability that is not significantly different from that of diclofenac sodium therapy at 3×25 mg per day. Recently, Bertorio [7] conducted research on the development of herbal therapy for osteoarthritis and demonstrated that the combination of ginger, soybean, and shrimp shell extracts provided significant results in reducing joint pain, stiffness, and physical disability. The findings were evaluated based on the Western Ontario and McMaster Universities Arthritis Index (WOMAC) and did not show a significant difference when compared with meloxicam.

Recently, research was conducted in several countries to evaluate the effectiveness of acupressure therapy for the pain of patients with osteoarthritis. The study by Alinaghizadeh et al [8] involved 40 patients with osteoarthritis who were divided into 2 groups (intervention and control). In the intervention group, 30 minutes of acupressure therapy was given for 5 days. The results showed that the average pain score in the intervention group decreased significantly, from 5.89 at the beginning to 4.11 at the end of the study, while the pain score did not change substantially in the control group. These findings remained consistent after adjusting for age, weight, and pretreatment covariates. This study supports the evidence that acupressure therapy provides an effective option for short-term knee pain relief in patients with knee osteoarthritis. In line with the results of this research, Akbarznezhad et al [9] conducted research on 51 older persons with osteoarthritis. The participants were divided into 3 groups (acupressure intervention, placebo, and controls). The findings of this study revealed that those who received acupressure therapy for 3-4 weeks, for 10-15 minutes each time, showed a significant reduction in the total WOMAC score, pain, and physical dysfunction.

Osteoarthritis with joint disfigurement causes continuous pain that results in increased medicine consumption, leading to a need to consider alternative complementary therapies for reducing pain [10]. Management of osteoarthritis still needs improvement. To date, no research has combined standardized curcuminoid turmeric extract therapy with acupressure for inflammation and pain in patients with osteoarthritis. More clinical trials with an appropriate methodology are needed to confirm the effectiveness of standardized turmeric extract, curcuminoids, and acupressure in treating physical problems in patients with osteoarthritis.

### Purpose

This study aims to investigate the efficacy of acupressure and standardized curcuminoids from turmeric extract to reduce inflammatory markers and pain, as well as endorphin hormones in the blood, while increasing quality of life in older patients with osteoarthritis genu.

## Methods

### Study Objectives

#### Primary Objective

This study aimed to determine the effectiveness of a combination of 2 regimens (acupressure and curcuminoid) as measured by changes from baseline in leukocytes, neutrophil leukocyte ratio, blood sedimentation rate, pain, secretion of COX-2, and endorphin hormones in the blood compared to placebo after 3 weeks of treatment in older patients with osteoarthritis.

#### Secondary Objectives

The secondary outcomes included pain, ability to perform activities of daily living, and quality of life. Pain was assessed with a visual analog scale. The ability to perform activities of daily living was assessed with the Barthel index and WOMAC. Quality of life was measured by respondents’ satisfaction in daily activities based on indicators in the Knee injury and Osteoarthritis Outcome Score (KOOS) instrument.

### Study Design

We conducted a 2-arm, double-blind (patient and investigational blinded) randomized controlled trial (RCT) to assess the efficacy, tolerability, and safety of the combination of acupressure and curcuminoid versus placebo.

### Prescreening

Patients were prescreened for specific X-ray and laboratory parameters. Following a screening visit, eligible participants started a washout period. After the washout period, eligible participants were randomized and treated for 3 weeks. The total duration of the study was 5 weeks.

### Study Setting and Source Population

Eligible participants were individuals recruited from communities covered by government-owned primary care hospitals who consulted a rheumatology subspecialist physician with symptoms of pain and discomfort around the knee.

Participants were recruited from August to October 2023. An informed consent form was provided to participants in their local language to gain credibility. To maintain the secrecy of data gathering, participant codes were provided, restricting access to only the lead researcher. After obtaining agreement from the participants, a thorough screening process was conducted to determine their eligibility.

### Population

The study population consisted of male and female patients (aged ≥65 years) with osteoarthritis (osteoarthritis with knee joint pain, knee joint stiffness in the morning for less than 30 minutes, crepitus, deformity, joint swelling [asymmetrically on the right and left], and other signs of inflammation [eg, a feeling of even warmth and reddish color]). The objective was to achieve a random allocation of about 70 patients. Given an anticipated screening failure rate of 25% and a washout failure rate of 20%, a total of about 100 patients underwent the screening procedures.

### Inclusion Criteria

In order to be considered for participation in this study, patients had to satisfy all of the following requirements: a clinical diagnosis of osteoarthritis that was confirmed by physical examination and X-rays; experience of pain with a numeric rating scale of 1-7; ability to swallow capsules; and mobility without assistance or with minimal assistance.

### Exclusion Criteria

Patients fulfilling the following criteria were not eligible for inclusion in this study (the investigator applied no additional exclusion criteria to ensure the study population represented all eligible patients): the presence of Parkinson disease, dementia, psychosis, new bone fractures, joint dislocations, cancer, rheumatic diseases other than osteoarthritis (eg, rheumatoid arthritis), and analgesic-dependent disease. Patients were also excluded if they were undergoing joint replacement therapy.

### Eligibility Test Procedure

Radiographs were used to assess participants for osteoarthritis using the Kellgren and Lawrence criteria, which categorize osteoarthritis from mild to severe. It should be noted that at the beginning of the disease, the radiographic appearance of the joint is still normal. According to Kellgren and Lawrence, radiologically, osteoarthritis is classified into 5 grades: grade 0 (normal), no signs of osteoarthritis; grade 1 (doubtful), without osteophytes, doubtful joint narrowing; grade 2 (minimal), few osteophytes on the tibia and patella and asymmetrical narrowing of the joint surface; grade 3 (moderate), moderate osteophytes present in several places, narrowing of the joint surface, and presence of subchondral sclerosis; and grade 4 (severe), presence of large osteophytes, complete narrowing of the joint surface, severe subchondral sclerosis, and joint surface damage.

The Mini Mental State Examination (MMSE) was used to assess the patient’s cognitive abilities, which is essential to ensure that a patient can understand the treatment and outcome measurement process instructions.

### Randomization, Allocation, and Blinding

**Figure 1:**
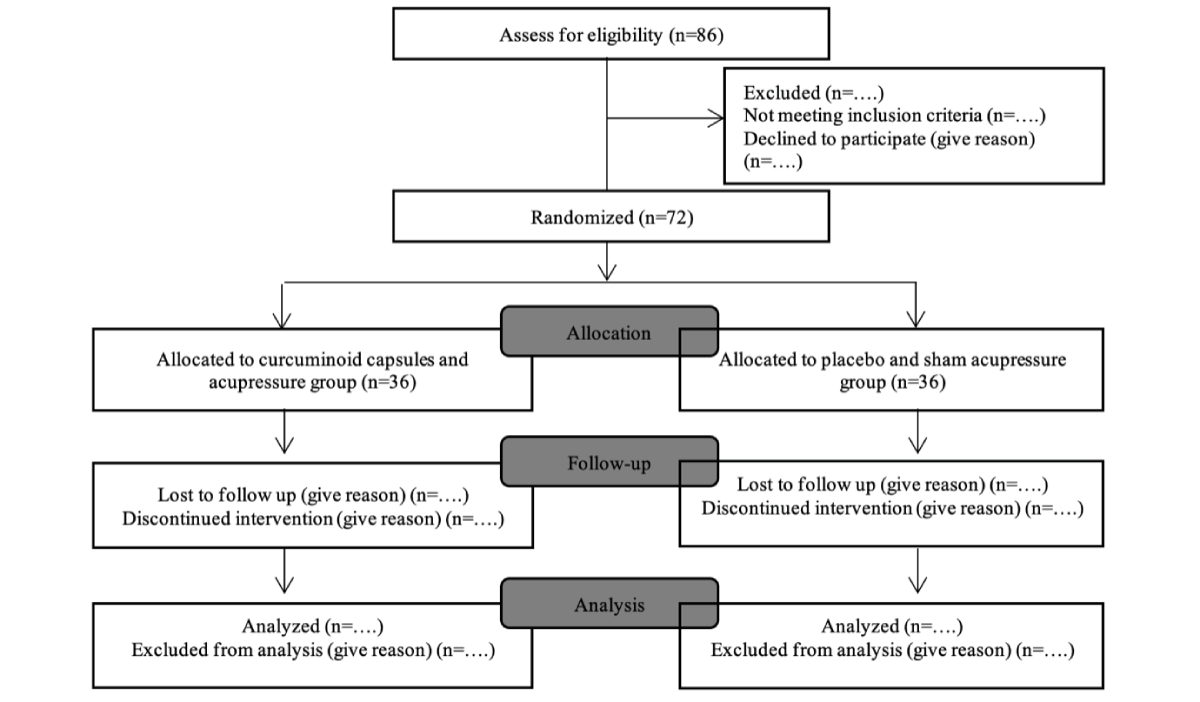
Schematic CONSORT (Consolidated Standards of Reporting Trials) flowchart for the study.

Seventy eligible participants were randomized to the group with curcuminoid capsules and acupressure (C+A; group 1) or the group with placebo and sham acupressure (P+S; group 2) via lottery randomization. Following this, the individuals were assigned randomly to either the C+A or P+S groups. Figure 1 displays the schematic CONSORT (Consolidated Standards of Reporting Trials) [11] flow diagram for the study procedure, as reported in the study.

The baseline characteristics for each participant were determined by the therapist using a standardized assessment form. During the measurement of baseline characteristics, various data were documented, including sex, age, body mass, pain characteristics, history of sickness, and medication use. The timetable of participant participation was developed in accordance with the SPIRIT (Standard Protocol Items: Recommendations for Interventional Trials) statement [12] (Table 1). The primary and secondary outcome indicators were assessed prior to administering the intervention.

**Table 1:** SPIRIT (Standard Protocol Items: Recommendations for Interventional Trials) recommended schedule for participation.

Timepoint			Enrollment		Allocation		Postallocation						Close-out						
					Week 0		Week 1		Week 2		Week 3		Day 1		Week 1		Week 1		After week 3
**Enrollment**																			
	Eligibility screening	✓																	
	Informed consent	✓																	
	Radiography and MMSE^a^ test	✓																	
	Allocation			✓															
**Interventions**																			
	C+A^b^ therapy					✓		✓		✓									
	P+S^c^ therapy					✓		✓		✓									
**Assessments**																			
	Demographic details	✓																	
	Inflammatory markers											✓						✓	
	Endorphins											✓						✓	
	Quality of life											✓		✓		✓		✓	

^a^MMSE: Mini Mental State Examination.

^b^C+A: curcuminoid capsules and acupressure.

^c^P+S: placebo and sham acupressure.

### Intervention

Following the administration of standardized evaluations and baseline measurements, the intervention was implemented. The participants were grouped randomly to either the C+A group or the P+S therapy group in a random manner. In the study, both the acupressure therapy and sham acupressure groups underwent a total of 2 sessions each week, each lasting 20 minutes, for a period of 3 consecutive weeks. During this period, participants were prohibited from regularly using any medications, with the exception of antihypertensive agents, thyroid medications, and antidiabetic therapies. The participants were provided with electronic message reminders in order to facilitate their adherence to the intervention. Preintervention session reminder texts were dispatched prior to each session. Each participant consumed curcuminoid capsules or placebo 3 times daily for a duration of 21 days.

**Figure 2:**
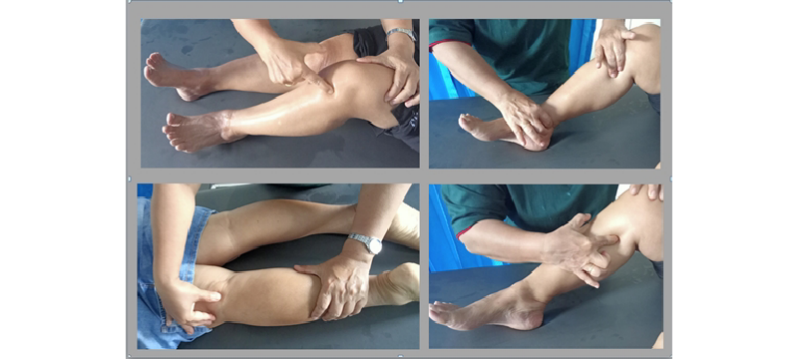
Acupressure massage points.

To perform acupressure in the C+A therapy group, the patient was put in the supine position with legs straight (Figure 2). The tools and materials were prepared, such as a seat (or bed), warm water, olive oil, wet and dry tissues, and towels. The therapist washed their hands, and the participant sat in a comfortable position. The patient was taught breathing relaxation techniques to use during therapy. The patient was allowed to pray according to their beliefs. To ensure the participant was relaxed and comfortable, some of the client’s clothing or accessories were removed if they might hinder the acupressure action being performed, if necessary. A client pain assessment was performed. The parts to be massaged were wiped with warm water treated with a disinfectant solution using a small towel, then dried with a clean towel. Using cream or oil, a warming massage was done with basic massage techniques, according to the client’s condition (using rubbing, squeezing, or pressing) and the patient’s feet were stretched. Acupressure massage was performed at the following points: ST34, ST36, *tai xi* acupoint, GB34, SP 9, and SP 8.

Sham acupressure was performed at 6 points in the P+S therapy group, all in the foot area, avoiding the therapeutic points used in the C+A therapy group.

The patient was asked to report any abnormal sensations or discomfort during the intervention. The researchers provided the study medications to the curcuminoid and placebo groups in a double-blind fashion. A standardized curcuminoid turmeric extract was prepared. Samples were first made from extracted turmeric and then optimized. The capsule formulation was made from turmeric rhizome extract, which contains 30 mg of curcuminoids per capsule. In the study, standardized curcuminoid turmeric extract was given in capsules 3 times a day for 3 weeks.

The researchers confirmed that the participants did not use any other intervention by performing a routine anamnesis that included visiting the participants at home 1 by 1 twice in a week, which was assisted by a village health worker (*kader*) and therapist.

### Study Outcomes

All outcome measures were assessed at baseline and after 3 weeks of intervention.

#### Primary Outcomes

##### Inflammatory Markers

Leukocyte numbers, the neutrophil lymphocyte ratio (NLR), and blood sediment rate were measured as inflammation markers. The examination used a complete blood test using a hematology analyzer, a laboratory tool used to measure and count the number of blood cells. The blood samples were mixed using a mixture of reagents to create a hemolyzing process. This process was divided into several parts for each purpose, that is, to measure leukocytes, neutrophils, platelets, and erythrocytes.

##### Secretion of the COX-2 Enzyme

The COX-2 enzyme is an essential mediator in increasing the inflammatory response. In the blood plasma of patients with osteoarthritis, the COX-2 enzyme can be detected. The COX-2 enzyme examination was conducted using the enzyme-linked immunosorbent assay (ELISA) method with blood plasma.

##### Secretion of Endorphin Hormones

Endorphins, in this case β endorphins, are hormones released by the pituitary gland in response to stress or pain, which are also secreted in blood plasma. Endorphin hormone levels in the blood were measured before starting therapy and after 3 weeks of therapy.

Endorphin hormone examination was done using the ELISA method on blood plasma that had been incubated at room temperature for 10-20 minutes, after which the tube was centrifuged for 20 minutes at a speed of 2000-3000 rpm.

#### Secondary Outcomes

The effectiveness of therapy is its ability to suppress other expected output indicators. Other output indicators were measured before starting therapy and after 3 weeks of therapy: (1) knee pain, as measured by the VAS score; (2) the WOMAC score, which assesses joint stiffness and joint functional ability; (3), the Barthel index; and (4) the KOOS.

WOMAC is a questionnaire developed by Bellamy et al [13] that continues to be developed to measure the functional abilities of patients with knee osteoarthritis. The WOMAC instrument has been translated into Indonesian. It has been tested for validity by Karsten et al [14] and has a Cronbach α coefficient of 0.966, indicating relatively high internal consistency. It is highly correlated, meaning that the WOMAC questionnaire in Indonesian is correct, validated, and can be used in Indonesian. This questionnaire evaluates 3 subscales by scoring on a 5-point ordinal scale: pain (5 items), stiffness (2 items), and physical function (17 items). The details of the scores are as follows: for pain, score 0 (no pain), score 1 (mild pain), score 2 (moderate pain), score 3 (severe pain), and score 4 (very severe pain); for stiffness, score 0 (not stiff), score 1 (mildly stiff), score 2 (medium stiffness), score 3 (extremely stiff), and score 4 (stiff to the point of locking); and for physical function, score 0 (not difficult), score 1 (somewhat difficult), score 2 (quite difficult), score 3 (very difficult), and score 4 (very difficult). The subscale score ranges are 0-20 for pain, 0-8 for stiffness, and 0-68 for physical function. The total score is obtained by adding up the scores of the 3 subscales, with a maximum score of 96. A higher WOMAC score indicates pain, stiffness, and worse physical function. The total WOMAC score can be categorized into 3 groups, namely low risk (score ≤60), moderate risk (score 60-80) and high risk (score ≥81).

The ability to perform activities of daily living in older people was assessed with the Barthel index questionnaire, which was first developed in 1965 by Mahoney and Barthel [15]. The Maryland State Medical Society holds the inventory of this instrument. In 2020, Pongantung et al [16] translated it into Indonesian and conducted a validity test, finding that interrater reliability was satisfactory. Regarding content validity, the Indonesian version of the Barthel index is acceptable. The construct validity test reveals 2 main factors: functional performance and physiological function.

Quality of life was assessed with the KOOS, an instrument to assess patient opinions about the condition of the knee and problems related to the knee. The KOOS has been translated into Indonesian and tested for validity by Phatama et al [17] with Cronbach α results of 0.84 to 0.97 for all subscales, indicating adequate internal consistency. The test-retest reliability demonstrates a high level of accuracy, as indicated by the intraclass correlation coefficients ranging from 0.91 to 0.99 across all subscales. There were no statistically significant variations observed in the replies of the KOOS subscales between the initial administration of the questionnaire and the subsequent administration within a span of 21 days. The validity and reliability of the Indonesian adaptation of the KOOS have been established, indicating that it serves as an objective tool for assessing knee ligament injuries and knee osteoarthritis in the Indonesian community. The KOOS can be used as a measurement of quality of life because it includes measurements of 5 subscales, namely, symptoms, rigidity, pain, daily activities, sports and recreation activities, and knee-related quality of life. The week prior to the test is the period considered when answering the questions. Answer options are standard (Likert scale), and each question is scored from 0 to 4. Scores are normalized (a score of 100 indicates no symptoms and 0 indicates extreme symptoms).

### Sample Size

The following formula was used to estimate the sample size:




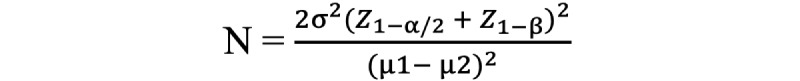




where *N* is the sample size required in both groups, and *s* is the SD of the primary outcome. This research has a confidence level (1–α) of 95%, so the value obtained is α=5%. Accordingly, the research hypothesis is unidirectional, so the magnitude of Z1–α=1.96. The research’s power (1–β) was also set at 80%, so if the value of β=20%, then the amount of Z1–β=0.842. Previous research [18] that analyzed changes in leukocytes in participants treated with curcuminoid therapy based on 30 mg turmeric extract taken 3 times a day for 14 days, amounting to 30.00±5.10 mg and meloxicam 1×15 mg per day, obtained a mean Δ value of leukocytes in the control group of 164.1 μL+50.91, and in the treatment group, 174.27 μL+78.93. The total number of participants required in the 2 groups was 60. Considering a dropout rate of 20%, the sample size was increased to 36 in each group.

### Ethical Considerations

The Medical and Health Research Ethics Committee (MHREC) of the Faculty of Medicine, Public Health and Nursing, Gadjah Mada University – Dr Sardjito General Hospital approved the study protocol, which was registered with the Forum for Ethical Review Committees in Asia and the Western Pacific (project number KE-FK-0674-EC-2023). The study was conducted according to Indonesia’s 2021 National Health Research and Development Ethical Guidelines and Standards by the Health Research and Development Ethics Committee and the Declaration of Helsinki, as revised in 2013. ClinicalTrials.gov has reviewed this protocol (NCT06105840).

Participation in this research was voluntary, without any coercion. All participants received comprehensive information about the research, especially about the intervention that would be given and the possible benefits and risks. Participants were allowed to withdraw at any time without penalty.

Participants were screened and provided a unique ID number to protect their personal information. The unique ID number was used to differentiate participants in an anonymous manner across measures and follow-up questionnaires. Participants who met the eligibility requirements of the initial screening form were prompted to fill out a contact form with their full name and address. Participants obtained benefits including an x-ray examination to determine the knee joint condition and therapy according to the research group they were assigned to. Participants were given tools for massage therapy (a towel and massage oil) that were theirs to keep. After the research activities were completed, participants in the control group received the same herbal therapy and acupressure as the treatment group. All information related to the identity of the research participants was kept secret and known only by the researchers, research staff, and auditor. Research results are published without the identity of the research participants.

### Adverse Events

We conducted an investigation of potential adverse events that could arise with the administration of the combined curcuminoid and acupressure therapy, encompassing symptoms such as heightened soreness, pain, numbness, and tingling. All instances of adverse events that pose a threat to life or were associated with a substantial impairment were duly reported to the ethics research committee of the institution. During the research, the researchers provided necessary protection in case of adverse events. This protection comprised examination by a physician and treatment in hospital.

### Data Analysis

The baseline characteristics of the participants will be assessed using descriptive statistics. The Shapiro-Wilk test will be used to determine the normality of the data gathered. Descriptive statistics will be presented using either the mean (SD) or the median (IQR), depending on the normality of the data. The Wilcoxon signed-rank test or a paired *t* test (2-tailed) will be used for within-group comparisons. In a similar vein, the Mann-Whitney *U* test or an independent *t* test (2-tailed) will be used to conduct comparisons between groups. The chosen level of significance for this study will be established at .05.

### Data Management

The collected data will be maintained in a secret manner for the duration of the study and disposed of after a period of 5 years. The process of collecting the initial data will involve the use of printed data-collection forms. These forms will afterward be organized and transcribed into an electronic format. The electronic data will be saved on a desktop computer that is not connected to the internet, as a precautionary measure to mitigate the risk of illegal access. This stored data will be used for further analysis. The individual appointed as the chair of the student project committee at the institute will be responsible for supervising data management.

## Results

In June 2023, the capsule formulation was prepared from turmeric rhizome extract, which had been tested and found to contain 30 mg of curcuminoids per capsule. The acupressure technique was formulated based on the therapeutic points recorded in the Acupressure Therapy Procedure Module to Improve Comfort in Knee Osteoarthritis, which has received a copyright from the Ministry of Law and Human Rights of the Republic of Indonesia with ID 000564960. The sham acupressure technique used points that had the potential to provide benefits and a relaxing effect in the feet but not at the main location of therapy. Therapeutic procedures and dosage were checked and approved by all authors.

In September 2023, a total of 84 older people who experienced joint pain declared themselves willing to be recruited. Physical examination and radiographs were used to assess the participants for osteoarthritis; 72 older people met the inclusion criteria. This allowed data analysis to take place in April 2024, and results should be submitted for publication in an international peer-reviewed journal by the end of 2024.

## Discussion

### Principal Findings

The benefits of herbs and acupressure can be helpful as additional options in treating inflammation and pain in patients with osteoarthritis. Curcuminoid is an active compound in turmeric that has natural anti-inflammatory properties. A meta-analysis conducted by Kou et al [19] showed that curcuminoid supplementation was effective in reducing clinical symptoms in the treatment of arthritis. The use of curcuminoid supplementation in patients with arthritis could reduce the possibility of unpleasant consequences. The results of systematic review showed that acupressure as a single or complementary intervention provides significant benefits in the management of osteoarthritis [10].

These studies showed that curcuminoid supplements may help reduce pain and inflammation in osteoarthritis. Acupressure points can stimulate the release of natural endorphins, which can help reduce pain and increase feelings of relaxation. Acupressure also works to help restore uninterrupted energy flow in the body, which can help reduce inflammation and improve joint function. Therefore, we deliberately chose an RCT design as it is often regarded as the preferred strategy for assessing and comparing the efficacy of therapies.

The strength of this study lies in its ability to prove the efficacy of a combination therapy for osteoarthritis with curcuminoids and acupressure. This is achieved through the use of an RCT design, which has never been used by other studies. This study uses an RCT because it can demonstrate the superiority of a new treatment over an existing standard treatment or a placebo. RCTs are the gold standard for ascertaining the efficacy and safety of a treatment. After conducting a systematic review [20], the researchers will continue their research using an RCT in the hope of obtaining the highest level of empirical evidence with control of the risk of systematic error (bias). Another advantage of this study is that the participants came from identical communities, so they are likely to have homogeneous demographics and characteristics, which is needed in RCTs.

### Limitations

The limitation of this study is that it does not consider the severity of osteoarthritis or the duration of osteoarthritis.

The main expected findings of the research are to determine biomarker changes, such as a decline in inflammatory markers as shown by leukocyte numbers and the NLR, a decline in the secretion of the COX-2 enzyme, and an increase in the secretion of endorphin hormones after conducting the intervention for 3 weeks. The secondary outcomes are the decline in knee pain, the increase in joint functional ability, and the increase in activities of daily living and quality of life.

Leukocyte count and the NLR are thought to increase linearly with the severity of osteoarthritis. The research of Taşoğlu et al [21] showed that a severe knee osteoarthritis group had a higher blood NLR than a mild to moderate group. A blood NLR of ≥2.1 was taken as the cutoff based upon the receiver operating characteristics (ROC). In the ROC curve analysis, a blood NLR ≥ 2.1 had 50% sensitivity and 77% specificity in predicting severe knee osteoarthritis. The results of this study suggest that blood NLR may be a novel and promising inflammatory marker indicating the severity of knee osteoarthritis. Blood NLR is a recent indicator of systemic inflammation. A blood NLR ≥2.1 is an independent risk factor for severe knee osteoarthritis.

This study seeks empirical evidence that COX-2 is expressed in peripheral blood as a result of the inflammatory process in patients with osteoarthritis. The anti-inflammatory action of curcuminoid-standardized turmeric extract is achieved by inhibiting the activity of cyclooxygenase and lipoxygenase, and also by acting as an antioxidant. In vitro curcuminoid can inhibit the activity of phospholipase, lipoxygenase, COX-2, leukotriene, prostaglandin, thromboxane, nitric oxide, collagenase, elastase, hyaluronidase, interferon, tumor necrosis factor α, and interleukin-12 [22].

In addition to COX-2 and NLR, the endorphin hormone was measured as a biomarker in this study. Pain changes experienced by people with osteoarthritis genu can be associated with secretions into the blood. The more endorphin enzymes are secreted by monocytes into the blood, the less pain will occur and the more comfort.

Knee pain in osteoarthritis is an unpleasant sensory and emotional experience due to potential or actual tissue damage. Pain is caused by the stimulation of chemical mediators such as histamine, bradykinin, acetylcholine, and prostaglandins. In addition to these substances that can stimulate pain sensitivity, the body also has substances that can inhibit pain. Endorphins and encephalin can relieve pain. Osteoarthritis pain is associated with severe inflammation and joint disfigurement. The more severe the inflammation and the joint changes, the more painful the joint becomes.

Joint stiffness and joint functional ability are related to pain and inflammation in osteoarthritis patients. The more severe the inflammation and pain, the more joint stiffness increases and joint functional ability declines. Pain is a significant factor in determining an individual’s perceived breakpoint, which in turn affects other aspects of life. The most prevalent symptom combinations are joint locking, knee swelling, and stiffness. Mobility limitation and the need for assistance increase as a result of pain and limited mobility. Another functional activity affected by limited functional mobility is the ability to perform self-care [23].

Quality of life is related to pain and the ability to be active. The more severe the pain and the greater the inability to perform daily activities, the lower the quality of life. The prolonged experience of pain in patients with osteoarthritis represents a breakpoint, a pivotal point in the experience of living with unrelenting pain, mobility limitations, and the subsequent consequences for physical and psychological well-being. These breakpoints experienced by patients with osteoarthritis are turning points for changes in quality of life. They are turning points that are precipitated by an increase in pain, the emergence of a combination of other symptoms, an increased limitation or loss of mobility, and changes in other functional activities [10].

### Conclusions

This document outlines a randomized clinical experiment that aims to examine and compare the impacts of curcuminoid and acupressure interventions on the amelioration of inflammation and pain and the enhancement of quality of life among older people diagnosed with osteoarthritis.

## Abbreviations

CAM: complementary alternative medicine
COX-2: cyclooxygenase-2
ELISA: enzyme-linked immunosorbent assay
KOOS: Knee injury and Osteoarthritis Outcome Score
NLR: neutrophil lymphocyte ratio
RCT: randomized controlled trial
ROC: receiver operating characteristic
WOMAC: Western Ontario and McMaster Universities Osteoarthritis Index

## Data Availability

The data sets generated during this study are available from the corresponding author on reasonable request.
